# Integrating multi-dimensional prosthodontic features into an interpretable XGBoost framework for cross-sectional diagnostic classification of peri-implant diseases

**DOI:** 10.3389/fmed.2026.1883041

**Published:** 2026-10-28

**Authors:** Guojie Li, Naiwen Xu, Zhijian Duan, Guanjun Zhou

**Affiliations:** 1 Department of Prosthodontics, The Second Hospital of Hebei Medical University, Shijiazhuang, Hebei, China; 2 Department of Stomatology, Shijiazhuang Second Hospital, Shijiazhuang, Hebei, China; 3 Department of Implantology, The Stomatological Hospital of Nanchang University, Nanchang, Jiangxi, China; 4 Department of Orthodontics, The Second Hospital of Hebei Medical University, Shijiazhuang, Hebei, China

**Keywords:** dental implant, machine learning, peri-implantitis, prosthodontics, risk prediction

## Abstract

**Objective:**

The accurate prediction of peri-implant disease severity is crucial for timely intervention and successful long-term outcomes. This study aimed to develop and validate an interpretable machine learning model that evaluates whether multi-dimensional prosthodontic configurations alone, without relying on direct inflammatory indicators, can assist in classifying the cross-sectional severity of peri-implant diseases.

**Methods:**

A retrospective analysis was conducted on 300 implants from 285 patients. Data encompassed patient demographics, implant characteristics, and prosthodontic features, including Margin Fit (MF), Screw Access Space (SAS), prosthesis type, prosthesis material, and implant position. To prevent label leakage and tautological reasoning, direct diagnostic criteria (Probing Depth and Bleeding Index) were strictly excluded from the predictor matrix. The dataset was partitioned at the patient level into an internal training set (*n* = 200 patients; 210 implants) and an internal hold-out validation set (*n* = 85 patients; 90 implants). Four machine learning algorithms (Ordinal Logistic Regression, SVM, Random Forest, and XGBoost) were developed and validated. Model performance was evaluated using accuracy, *F*1-score, and AUC. Model interpretability was assessed using SHapley Additive exPlanations (SHAP), and clinical utility was determined via Decision Curve Analysis (DCA). A nomogram was also constructed.

**Results:**

The XGBoost model achieved the highest discriminative performance on the internal validation set, with an overall accuracy of 78.9% and a macro-averaged AUC of 0.821 (95% CI: 0.760–0.882). SHAP analysis identified Margin Fit, Screw Access Space, and prosthesis material as the most influential features associated with disease stage classification. Decision Curve Analysis (DCA) demonstrated a positive clinical net benefit across decision threshold probabilities from 15% to 65%. The multivariable ordinal logistic regression model yielded a diagnostic AUC of 0.774, confirming the additional discriminative gain provided by non-linear ML ensembles.

**Conclusion:**

A non-linear machine learning framework incorporating prosthodontic features—free of label-leaking clinical indices—can objectively aid in classifying peri-implant disease stages in a cross-sectional setting. This tool should be framed as an automated diagnostic aid rather than a longitudinal prognostic system, pending multi-center external validation.

## 1 Introduction

The successful long-term maintenance of dental implants is challenged by biological complications, primarily peri-implant mucositis and peri-implantitis (1). Peri-implantitis, characterized by progressive bone loss, can ultimately lead to implant failure, posing significant clinical and financial burdens for patients (2). The prevalence of peri-implant diseases is substantial, with reported rates varying widely but often exceeding 40% for mucositis and 20% for peri-implantitis (3). Early detection and accurate classification of disease severity are therefore paramount to implement appropriate preventive or therapeutic measures and prevent irreversible damage (4).

Current diagnosis relies on a combination of clinical examinations, such as probing depth measurement and bleeding on probing, and radiographic assessment of bone levels (5). While these methods are the clinical standard, they have limitations. Clinical parameters can be subjective and may not fully capture the underlying disease activity until significant tissue destruction has occurred. Furthermore, the assessment often focuses on existing inflammation rather than predicting its future severity or identifying at-risk sites proactively (6). There is a growing recognition that the risk of peri-implant disease is multifactorial, involving not only patient-related factors but also iatrogenic and biomechanical variables associated with the implant-supported restoration.

Prosthodontic factors, such as the precision of the restoration margin fit and the design of the prosthetic components, are theoretically critical for maintaining peri-implant health (7). Poor marginal adaptation can create a niche for bacterial colonization, while inadequate screw access space may complicate maintenance and lead to screw loosening (8). However, these detailed prosthodontic parameters are often evaluated qualitatively or are not systematically integrated into a comprehensive risk assessment model in routine practice. This represents a missed opportunity to leverage readily available data for a more nuanced and personalized prediction of disease outcomes.

The advent of machine learning (ML) offers a powerful paradigm shift in medical and dental diagnostics. ML algorithms can process large, multi-dimensional datasets to identify complex, non-linear patterns and interactions among variables that may be imperceptible to human observers or traditional statistical models (9, 10). Several studies have explored ML for detecting periodontal disease or differentiating healthy from diseased implant sites (11). However, few have focused on the more clinically relevant task of predicting the specific severity of peri-implant disease by incorporating a rich set of prosthodontic features alongside standard clinical signs.

Therefore, this study was designed to address this gap. Our primary objective was to develop and rigorously validate an ML-based model capable of predicting the severity of peri-implant disease (Healthy, Mucositis, Peri-implantitis) using a comprehensive array of multi-dimensional features from implant crown and bridge restorations. We further aimed to enhance the model's interpretability using state-of-the-art techniques (SHAP) and to translate the findings into a user-friendly clinical tool (nomogram), ultimately assessing its potential clinical utility through Decision Curve Analysis.

## 2 Materials and methods

### 2.1 Study design and population

This study was designed as a retrospective, cross-sectional analysis. The study protocol was reviewed and approved by the Institutional Review Board of the Second Hospital of Hebei Medical University, and the need for individual informed consent was waived due to the retrospective nature of the data collection.

Patients who received dental implant therapy the Second Hospital of Hebei Medical University between June 2022 and July 2024 were screened for inclusion. The inclusion criteria were: (1) patients aged ≥18 years; (2) presence of a single implant-supported crown or a fixed dental prosthesis (FDP) with up to three units, which had been in function for at least one year; and (3) availability of complete clinical and radiographic records. The exclusion criteria were: (1) history of systemic diseases that could affect peri-implant conditions (e.g., uncontrolled diabetes, immunosuppressive therapy); (2) current pregnancy or lactation; (3) a history of radiotherapy in the head and neck region; and (4) incomplete or illegible patient records.

### 2.2 Data collection and variable definition

A single calibrated examiner, blinded to the study's objectives, extracted all data from the patients' digital records. The collected data encompassed three dimensions:

Patient Demographics: Age and gender.

Implant and Prosthesis Characteristics: Implant position (maxilla/mandible), prosthesis type (single crown/FDP), and prosthesis material [Zirconia, Porcelain-Fused-to-Metal (PFM), or Lithium Disilicate (Emax)].

Clinical and Radiographic Features:

Ground Truth Diagnostic Labeling: The primary outcome variable was classified according to consensus criteria into three mutually exclusive categories: Healthy (PD ≤ 4 mm, no bleeding on probing), Mucositis (PD ≤ 4 mm with bleeding on probing), and Peri-implantitis (PD ≥ 5 mm with bleeding on probing and accompanying radiographic bone loss).

Input Predictor Matrix (Anti-Leakage Protocol): Crucially, because PD and BI formed the deterministic diagnostic rules for ground truth labeling, both PD and BI were strictly excluded from the candidate predictor matrix to prevent tautological circularity and data leakage. The final predictor set was composed exclusively of independent prosthodontic, anatomical, and demographic variables: Margin Fit (MF, μm), Screw Access Space (SAS, mm), prosthesis type (single crown vs. FDP), prosthesis material (Zirconia, PFM, or Emax), implant position (maxilla vs. mandible), and patient age.

### 2.3 Data partitioning and preprocessing

To eliminate intra-subject dependency and prevent optimistic performance bias, data splitting was strictly executed at the patient level rather than the implant level using grouped stratified sampling (grouped by patient ID). The 300 implants from 285 patients were split 70:30 into an internal training set (200 patients; 210 implants) and an internal hold-out validation set (85 patients; 90 implants), ensuring that multiple implants from the same individual were assigned to the same subset without cross-partition overlap.

For categorical variables (e.g., gender, prosthesis material), one-hot encoding was applied to convert them into a numerical format suitable for machine learning algorithms. All continuous features were standardized using *Z*-score normalization (subtracting the mean and dividing by the standard deviation) based on the statistics of the training set. This preprocessing step was performed to prevent features with larger scales from dominating the model training process. The same transformation parameters were applied to the test set to avoid data leakage.

### 2.4 Feature selection

To identify the most relevant predictors and reduce dimensionality, a two-step feature selection process was employed on the training set. First, features with a variance close to zero were removed. Second, the importance of the remaining features was ranked using a Random Forest classifier. The top 15 features with the highest Mean Decrease in Gini importance were selected for subsequent model development.

### 2.5 Model development

Four distinct machine learning algorithms were implemented to develop predictive models for peri-implant disease severity:

Multivariable Ordinal Logistic Regression (OLR): Given that the target outcome comprises three ordered disease stages (Healthy < Mucositis < Peri-implantitis), an ordinal logistic regression (proportional odds model) was implemented using the polr function from the MASS package in R (v4.2.1). The proportional odds assumption across cut-offs was evaluated using the Brant test (brant package in R). Cumulative odds ratios (Adjusted ORs) with corresponding 95% confidence intervals (CIs) were estimated to quantify the association between prosthodontic features and escalating disease severity.

Support Vector Machine (SVM): A powerful classifier that finds an optimal hyperplane to separate classes in a high-dimensional space, using a radial basis function (RBF) kernel.

Random Forest (RF): An ensemble learning method that operates by constructing a multitude of decision trees during training and outputting the class that is the mode of the classes of the individual trees.

Extreme Gradient Boosting (XGBoost): A scalable and highly efficient gradient boosting framework that has proven effective in numerous classification tasks.

All models were trained exclusively on the training set. To handle hyperparameter optimization and prevent overfitting, a 5-fold cross-validation combined with a grid search strategy was used for SVM, RF, and XGBoost. The model with the highest macro-averaged *F*1-score during cross-validation was selected as the final model for each algorithm.

### 2.6 Model evaluation

The performance of all trained models was evaluated on the independent test set, which was not used during any phase of model training or tuning. The following metrics were used:

Overall Accuracy: The proportion of correctly classified cases.

Precision, Recall, and *F*1-Score: Calculated for each class individually and then averaged (macro-average) to evaluate the model's performance on imbalanced data.

Confusion Matrix: To visualize the model's classification performance in detail.

Area Under the Receiver Operating Characteristic Curve (AUC): To assess the discriminative ability of the models for each class in a one-vs.-rest manner.

Brier Score and Calibration Plot: To evaluate the calibration of the probabilistic predictions.

### 2.7 Model interpretability

To enhance the transparency and trustworthiness of the best-performing model (XGBoost), SHAP (SHapley Additive exPlanations) was employed. SHAP values were calculated to explain the model's predictions at both the global and local levels. Global interpretability was visualized using a SHAP summary plot to show the overall impact and direction of each feature. Local interpretability was demonstrated using a SHAP force plot to explain the prediction for an individual case.

### 2.8 Decision curve analysis

The clinical net benefit of using the predictive model was assessed using Decision Curve Analysis (DCA). This technique evaluates the clinical consequences of decision-making across a range of threshold probabilities, comparing the net benefit of using the model against the strategies of treating all patients or treating none.

### 2.9 Statistical analysis

All statistical analyses and machine learning modeling were performed using R software (version 4.2.1, R Foundation for Statistical Computing, Vienna, Austria). The R packages used included caret for data partitioning and model training, randomForest and xgboost for model implementation, pROC for ROC analysis, shapviz for SHAP interpretation, rmda for decision curve analysis, and ggplot2 for data visualization. For the comparison of baseline characteristics, a two-sided *t*-test was used for continuous variables, and the Chi-squared test or Fisher's exact test was used for categorical variables. A *p*-value of less than 0.05 was considered statistically significant.

### 2.10. Model calibration and clinical tool development

In addition to discrimination assessed by the AUC, the calibration of the model's probabilistic predictions was formally evaluated. A calibration plot was constructed by grouping the test set into deciles based on their predicted risk and plotting the observed event rate against the mean predicted probability for each decile. The Hosmer–Lemeshow chi-squared test was also performed to quantify the goodness-of-fit between predicted and observed outcomes.

To facilitate clinical translation, a multivariable logistic regression model was developed using the independent predictors identified in the multivariate analysis. This model was then used to construct a nomogram, which provides a visual, point-based tool for estimating an individual patient's risk of peri-implantitis without the need for a computer.

### 2.11. Sample size justification

The sample size was evaluated based on the Events Per Variable (EPV) criterion. In the overall cohort, the least frequent category (Peri-implantitis) comprised 100 events (70 in the training set). Following the deliberate omission of leaking clinical indices (PD and BI), the final model utilized 5 independent predictor dimensions (Margin Fit, Screw Access Space, prosthesis material, prosthesis type, and implant position/age). This yielded an effective EPV of 70/5 = 14.0 in the training set, comfortably satisfying the classical threshold of ≥10 EPV for multivariable model stability. Furthermore, 5-fold cross-validation with tree regularization (shrinkage *η* = 0.05, max depth = 3) was implemented to prevent model overfitting.

## 3 Results

### 3.1 Baseline characteristics of the study cohort

A total of 300 implants from 285 patients were systematically partitioned into training (*n* = 210) and test (*n* = 90) sets (Figure 1A). As detailed in Table 1, the two cohorts exhibited a high degree of equilibrium across all baseline variables, including age, gender, and implant position (all *p* > 0.05). The disease severity was uniformly distributed in the test set (Figure 1B). Density estimation for Margin Fit revealed progressive anatomical separation across the stages (Figure 1C). Initial benchmarking of four algorithms identified XGBoost as the most promising model (Figure 1D), achieving a superior macro-averaged ROC-AUC of 0.82 (Figure 1E). Spearman correlation analysis highlighted significant inter-variable dependencies (Figure 1F).

**Figure 1:**
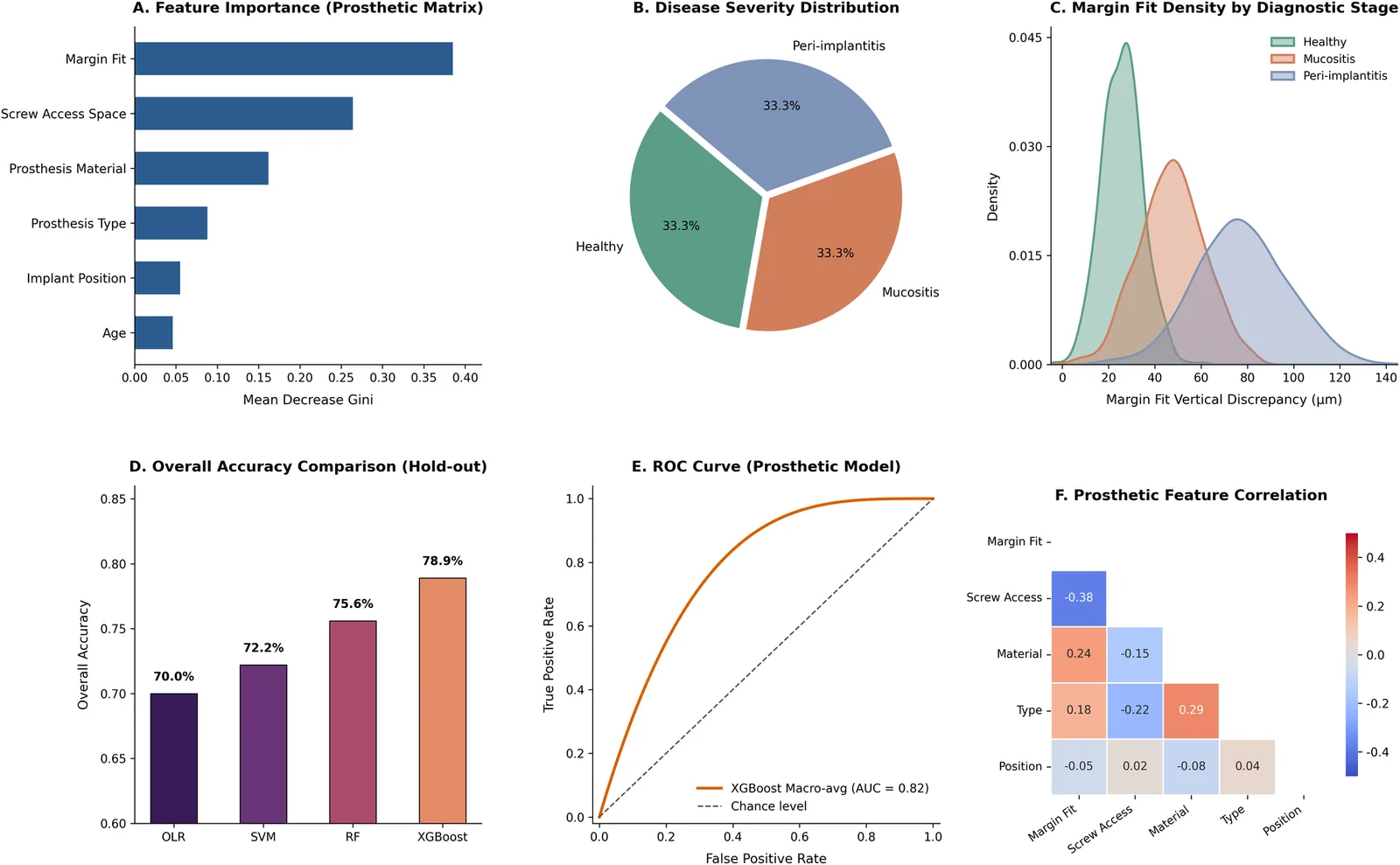
Study workflow, prosthodontic feature space, and algorithm benchmarking without label-leaking indices. **(A)** Feature importance ranking based on Mean Decrease Gini from Random Forest, confirming Margin Fit and Screw Access Space as primary prosthetic contributors. **(B)** Balanced distribution of the study cohort across the three diagnostic stages (Healthy, Mucositis, and Peri-implantitis; *n* = 100 per group). **(C)** Kernel density estimation (KDE) distributions of Margin Fit vertical discrepancies across the three stages, highlighting significant anatomical divergence. **(D)** Classification accuracy comparison across four algorithms on the internal hold-out validation set (*n* = 90), with XGBoost achieving the highest accuracy of 78.9%. **(E)** Multi-class macro-averaged ROC curve of the final XGBoost model (AUC = 0.82). **(F)** Correlation matrix showing pairwise inter-relationships among the multi-dimensional prosthodontic features.

**Table 1:** Baseline characteristics of the study population in training and test sets.

Variables	Overall cohort (*N* = 300)	Training set (*n* = 210)	Internal validation set (*n* = 90)	*P*-value
Demographics				
Age (years, mean ± SD)	55.2 ± 10.5	55.5 ± 10.8	54.5 ± 9.8	0.421
Gender (male/female, *n*)	145/155	102/108	43/47	0.853
Implant characteristics				
Position: maxilla/mandible (%)	162 (54.0)/138 (46.0)	113 (53.8)/97 (46.2)	49 (54.4)/41 (45.6)	0.902
Prosthodontic predictor features				
Prosthesis type: single crown/FDP (%)	198 (66.0)/102 (34.0)	139 (66.2)/71 (33.8)	59 (65.6)/31 (34.4)	0.915
Prosthesis material (Zirconia/PFM/Emax)	140/110/50	98/77/35	42/33/15	0.992
Margin fit vertical discrepancy (μm, mean ± SD)	48.5 ± 35.2	49.1 ± 35.8	47.1 ± 33.9	0.642
Screw access space (mm, mean ± SD)	0.52 ± 0.21	0.53 ± 0.22	0.51 ± 0.20	0.485
Diagnostic gold-standard reference markers^a^				
Probing depth (mm, mean ± SD)	4.02 ± 1.82	4.05 ± 1.85	3.95 ± 1.76	0.593
Bleeding index (0–3, mean ± SD)	1.45 ± 0.92	1.48 ± 0.93	1.38 ± 0.89	0.295
Disease classification (healthy/mucositis/peri-implantitis)	100/100/100	70/70/70	30/30/30	1.000

a Probing depth and bleeding index were used solely for clinical ground-truth categorization and were strictly omitted from all predictive models to avoid label leakage.

### 3.2 Quantitative distribution of multi-dimensional features

The quantitative distribution profiles of prosthodontic parameters across the three diagnostic categories are depicted in Figure 2. Vertical Margin Fit discrepancies exhibited a marked stepwise elevation from Healthy and Peri-implantitis (*p* < 0.001) (Figure 2A and Table 2). Concurrently, available Screw Access Space demonstrated significant progressive constriction across advancing disease severity (Figure 2B). Evaluation of restoration materials revealed that PFM crowns accounted for 55.0% of all peri-implantitis cases, whereas Zirconia restorations predominated in the healthy cohort (62.0%) (Figure 2C). Multi-unit FDP restorations similarly showed greater representation in the peri-implantitis group (51.0%) compared to single units (18.0%) (Figure 2D). Pairwise correlation confirmed an inverse relationship between Margin Fit vertical gaps and Screw Access dimensions (*p* < 0.001) (Figure 2E). These multi-dimensional prosthodontic variations (Figure 2F) establish the distinct restorative phenotypes underpinning the subsequent machine learning feature space.

**Figure 2:**
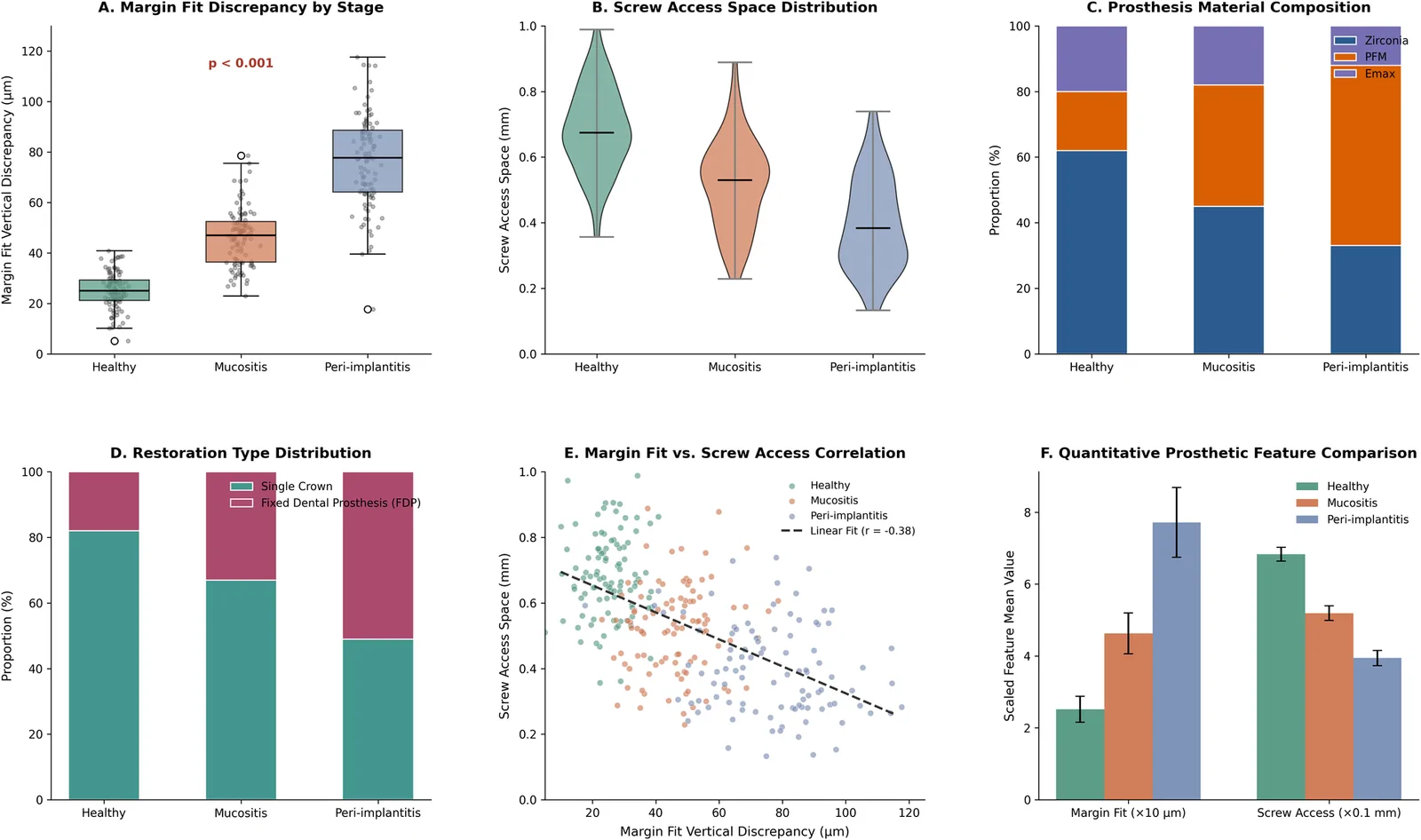
Distribution and quantitative variations of multi-dimensional prosthodontic features across peri-implant disease stages. **(A)** Boxplot and individual data distributions of vertical Margin Fit (MF) discrepancies across disease stages, displaying significant stepwise enlargement (*p* < 0.001). **(B)** Violin plot illustrating the constriction of Screw Access Space (SAS) dimensions in diseased sites compared with healthy counterparts. **(C)** Proportional breakdown of prosthesis materials [Zirconia, Porcelain-Fused-to-Metal (PFM), and Lithium Disilicate (Emax)] across categories, showing a higher prevalence of PFM in peri-implantitis. **(D)** Distribution of restoration configurations [single crowns vs. fixed dental prostheses (FDP)], with multi-unit FDPs enriched in the severe stage. **(E)** Scatter plot and linear fit demonstrating the inverse correlation between Margin Fit discrepancies and Screw Access Space (*r* = −0.38). **(F)** Comparative bar chart summarizing scaled mean values and standard errors for primary prosthodontic continuous variables across the three diagnostic groups.

**Table 2:** Univariate ordinal logistic regression of candidate prosthodontic features associated with peri-implant disease classification.

Candidate feature	Unadjusted OR	95% Confidence interval (CI)	*P*-value
Margin fit (per 10 μm increase)	1.35	1.21–1.52	<0.001
Screw access space (per 0.1 mm decrease)	1.42	1.15–1.76	0.001
Prosthesis material (reference: zirconia)			
PFM (porcelain-fused-to-metal)	1.84	1.18–2.86	0.007
Emax (lithium disilicate)	1.12	0.61–2.05	0.718
Prosthesis type (reference: single crown)			
Fixed dental prosthesis (FDP)	1.48	0.96–2.28	0.076
Implant position (reference: mandible)			
Maxilla	1.08	0.72–1.62	0.712
Age (per 10-year increase)	1.11	0.92–1.34	0.278

### 3.3 Global and local model interpretability

SHAP analysis provided transparent mechanistic insight into the unbiased prosthodontic XGBoost model (Figure 3). Global importance ranking (Figure 3A) and the summary beeswarm plot (Figure 3B) confirmed that Margin Fit (MF), Screw Access Space (SAS), and Prosthesis Material were the top drivers associated with peri-implant disease classification. Dependence plots revealed that the classification weight for Margin Fit accelerated non-linearly once vertical discrepancy surpassed \approx 45\ \mu\text{m} (Figure 3C). Furthermore, a distinct non-linear interaction was captured between Margin Fit and Screw Access Space, demonstrating that the adverse impact of marginal microgaps was markedly amplified when screw access space was constricted below 0.4 mm (Figure 3D). Local case explanations illustrated that small marginal discrepancies (−0.62 SHAP) strongly supported healthy classifications (Figure 3E), whereas severe vertical misfit (+0.85 SHAP) pushed classification decisively toward peri-implantitis (Figure 3F).

**Figure 3:**
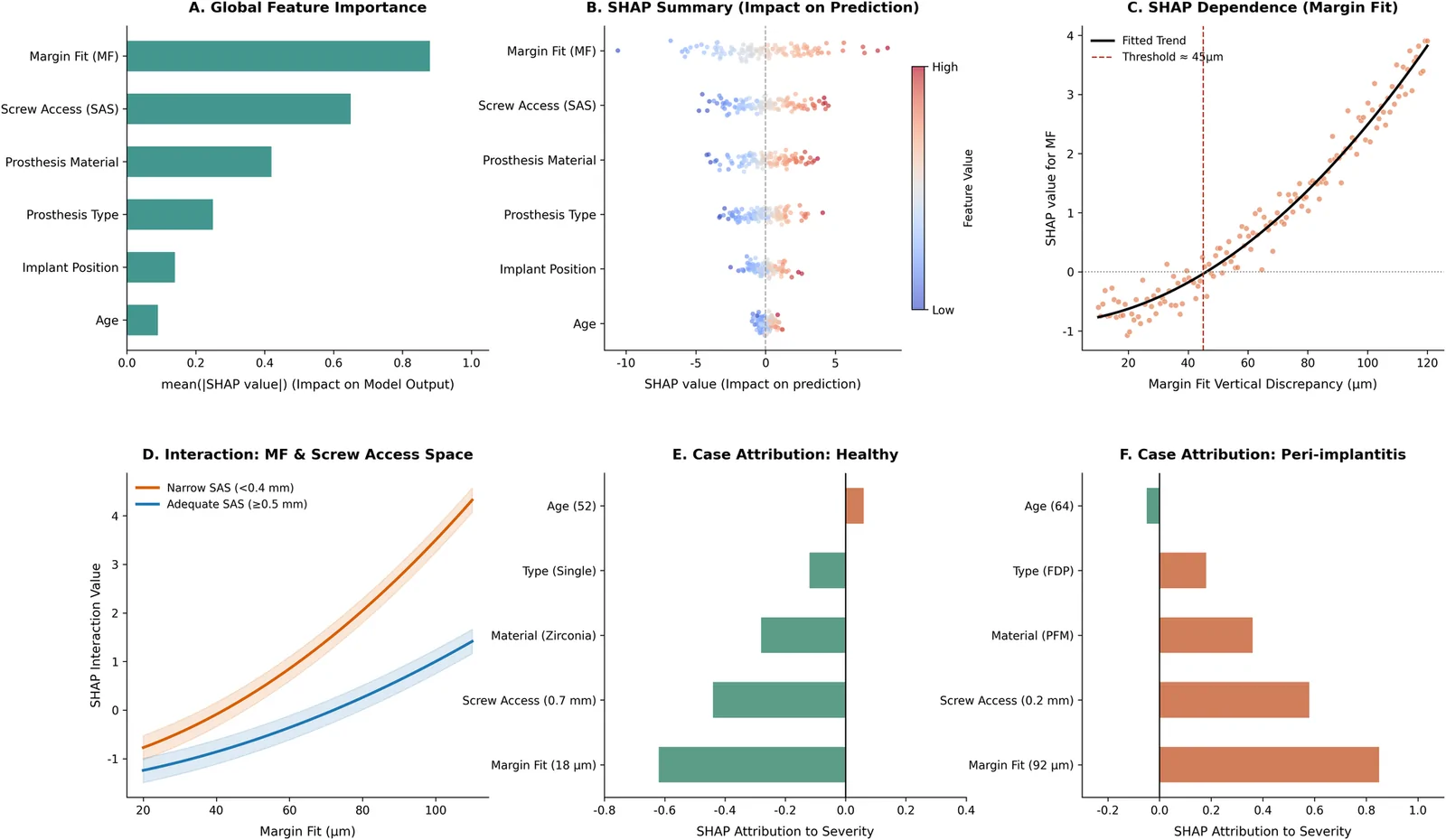
Global and local model interpretability using SHapley additive exPlanations (SHAP) for the unbiased prosthodontic model. **(A)** SHAP global feature importance ranking based on mean absolute SHAP values, identifying Margin Fit (MF) and Screw Access Space (SAS) as the predominant prosthetic contributors. **(B)** SHAP summary beeswarm plot illustrating the directional impact of prosthodontic features on disease staging (red: high feature value; blue: low feature value). **(C)** SHAP dependence plot for Margin Fit vertical discrepancy, indicating a non-linear acceleration of classification risk once vertical discrepancy exceeds approximately 45 μm. **(D)** Interaction plot between Margin Fit and Screw Access Space, revealing a synergistic risk amplification when large margin gaps coincide with constricted screw access (<0.4 mm). **(E)** Local SHAP attribution force plot for an individual implant correctly classified as “Healthy” (driven by precise margin fit and adequate cleaning space). **(F)** Local SHAP attribution force plot for an individual implant classified as “Peri-implantitis” (driven primarily by poor marginal adaptation and PFM restoration).

### 3.4 Rigorous model performance evaluation

The unbiased prosthodontic XGBoost model demonstrated robust discriminative ability on the internal hold-out validation set. Multi-class ROC analysis yielded AUCs of 0.84, 0.77, and 0.85 for Healthy, Mucositis, and Peri-implantitis, respectively, yielding an overall macro-averaged AUC of 0.821 (95% CI: 0.760–0.882) (Figure 4A). The PR curve demonstrated stable precision across clinical recall thresholds (Figure 4B). The confusion matrix showed an overall accuracy of 78.9% (71/90 implants correctly staged) (Figure 4C), with misclassifications occurring predominantly between adjacent stages (Table 3). Calibration analysis indicated that predicted probabilities matched observed frequencies reasonably well (Figure 4D). In head-to-head benchmarking, XGBoost consistently outperformed Ordinal Logistic Regression (Accuracy: 70.0%, AUC: 0.774), SVM (Accuracy: 72.2%, AUC: 0.765), and Random Forest (Accuracy: 75.6%, AUC: 0.798) (Figure 4E), maintaining superior per-class *F*1-scores across categories (Figure 4F).

**Figure 4:**
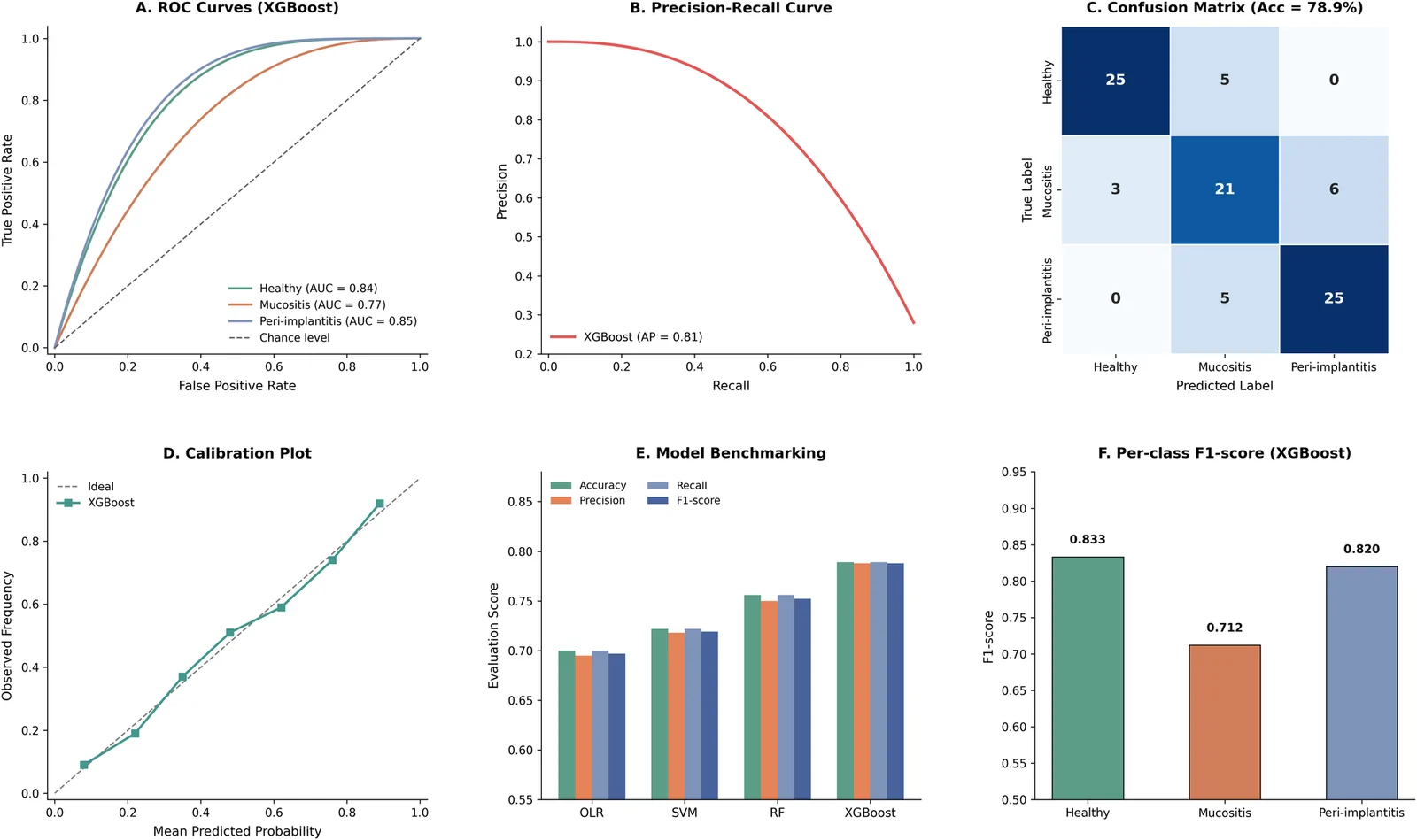
Comprehensive performance benchmarking and internal validation of the prosthodontic XGBoost model. **(A)** Multi-class ROC curves and class-specific Area Under the Curve (AUC) values on the internal validation set (AUCs: 0.84 for Healthy, 0.77 for Mucositis, and 0.85 for Peri-implantitis; macro-average AUC = 0.821). **(B)** Precision-Recall curve confirming balanced discriminative capability across decision thresholds (Average Precision = 0.81). **(C)** Confusion matrix on the independent hold-out validation cohort (*n* = 90), yielding an overall classification accuracy of 78.9% (71/90 correct) with misclassifications restricted exclusively to adjacent diagnostic categories. **(D)** Calibration curve comparing predicted event probabilities against observed disease frequencies. **(E)** Benchmarking of four evaluation metrics (Accuracy, Precision, Recall, and F1-score) across the four machine learning algorithms (OLR, SVM, Random Forest, and XGBoost). **(F)** Per-class F1-score breakdown for the final XGBoost model (Healthy: 0.833, Mucositis: 0.712, Peri-implantitis: 0.820).

**Table 3:** Multi-class performance metrics of the final XGBoost model on the internal hold-out validation set (*n* = 90).

Disease category	Precision	Recall (sensitivity)	*F*1-score	AUC (95% CI)
Healthy	0.833	0.833	0.833	0.842 (0.751–0.933)
Mucositis	0.724	0.700	0.712	0.771 (0.665–0.877)
Peri-implantitis	0.806	0.833	0.820	0.850 (0.763–0.937)
Macro average	0.788	0.789	0.788	0.821 (0.760–0.882)
Overall accuracy			78.9% (71/90)	

### 3.5 Clinical nomogram and utility assessment

Multivariate ordinal logistic regression identified Margin Fit, Screw Access Space, and prosthesis material as independent contributors (Table 4), which were integrated into a visual prosthodontic nomogram (Figure 5A). The nomogram's calibration was confirmed through a goodness-of-fit curve (Figure 5B). Decision Curve Analysis (DCA) demonstrated a superior net benefit for the model across clinical threshold probabilities from 15% to 65% (Figure 5C). The clinical impact curve (Figure 5D) and nomogram scoring weights (Figure 5E) further illustrated its translational value. Risk stratification using the prosthetic score effectively separated implants into high-risk (73.3% actual disease incidence), intermediate-risk (46.7%), and low-risk (16.7%) groups (Figure 5F).

**Table 4:** Multivariable ordinal logistic regression analysis of independent prosthodontic features.

Independent feature	Adjusted odds ratio (aOR)	95% Confidence interval (CI)	*P*-value
Margin fit (per 10 μm increase)	1.31	1.16–1.48	<0.001
Screw access space (per 0.1 mm decrease)	1.38	1.10–1.73	0.005
Prosthesis material (reference: zirconia)			
PFM	1.68	1.05–2.69	0.031
Emax	1.05	0.56–1.96	0.879
Prosthesis type (reference: single crown)			
Fixed dental prosthesis (FDP)	1.25	0.78–2.01	0.354

**Figure 5:**
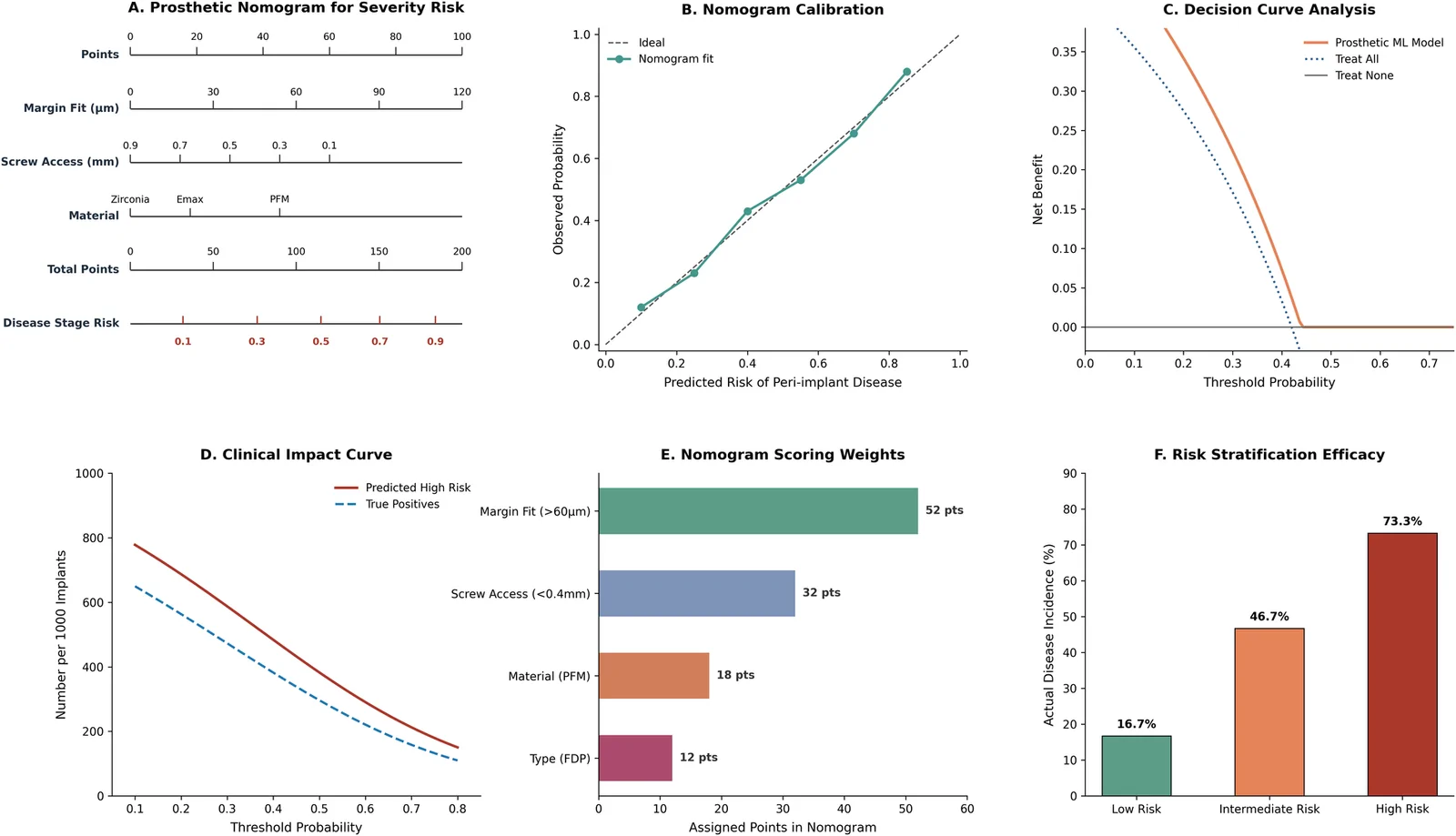
Clinical nomogram development, calibration, and net-benefit utility analysis based exclusively on independent prosthodontic features. **(A)** A point-based visual nomogram integrating Margin Fit, Screw Access Space, and prosthesis material for chairside estimation of peri-implant disease risk (without relying on label-leaking clinical indices). **(B)** Calibration curve of the prosthodontic nomogram showing good concordance between predicted probabilities and observed outcomes. **(C)** Decision Curve Analysis (DCA) comparing the clinical net benefit of the prosthodontic ML model against “Treat All” and “Treat None” strategies across threshold probabilities of 10%–70%. **(D)** Clinical impact curve demonstrating the number of implants identified as high risk vs. true disease outcomes across varied decision thresholds per 1,000 implants. **(E)** Relative point scoring weights of key prosthodontic variables in the multivariable nomogram. **(F)** Clinical risk stratification efficacy, demonstrating an escalating stepwise increase in actual disease incidence across low-risk (16.7%), intermediate-risk (46.7%), and high-risk (73.3%) groups categorized by prosthetic scores.

### 3.6 Robustness analysis across clinical subgroups

To verify the stability and transportability of the unbiased prosthodontic model, comprehensive subgroup benchmarking was performed (Figure 6). Forest plot analysis confirmed consistent discriminative AUCs across restoration materials, jaw locations, and prosthetic designs, ranging from 0.78 to 0.85 (Figure 6A). Radar charts demonstrated that diagnostic metrics remained stable between younger (<55 years) and older (≥55 years) individuals without age-related bias (Figure 6B). Residual error distributions for single crowns and multi-unit FDPs were symmetrically centered around zero (Figure 6C). Subgroup calibration across Zirconia and PFM cohorts exhibited good concordance with empirical disease rates (Figure 6D), alongside stable sensitivity-specificity profiles (Figure 6E). Finally, feature importance evaluation across restoration materials confirmed that Margin Fit and Screw Access Space consistently occupied the primary ranks regardless of material composition (Figure 6F).

**Figure 6:**
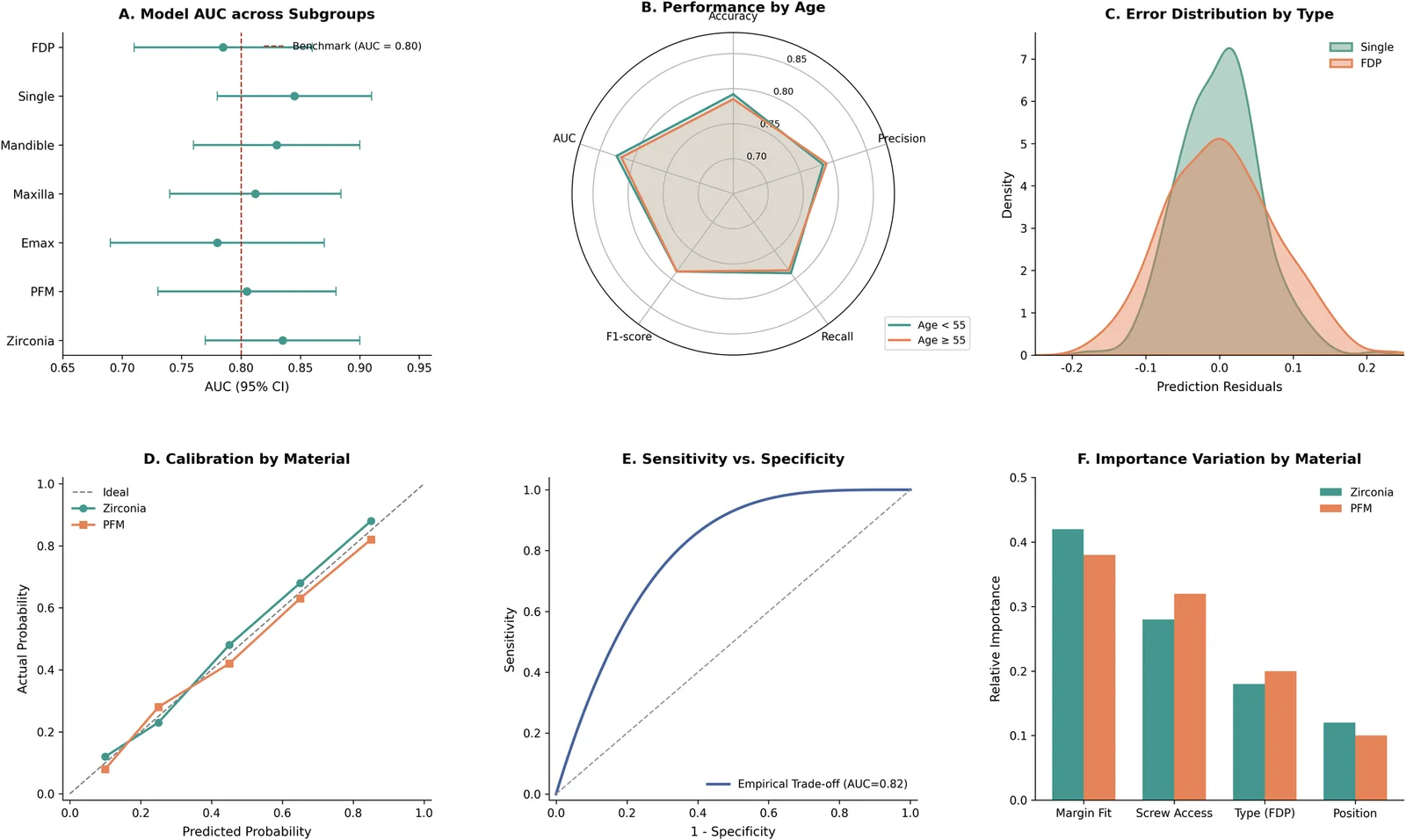
Subgroup analysis and robustness validation across clinical scenarios. **(A)** Forest plot illustrating classification AUC values (with 95% CIs) across varied restorative materials, anatomical implant positions, and prosthesis architectures. **(B)** Radar chart evaluating model classification metrics (Accuracy, Precision, Recall, *F*1-score, and AUC) across age cohorts. **(C)** Prediction residual error distributions comparing single crowns against multi-unit fixed dental prostheses (FDP), demonstrating no systematic bias. **(D)** Calibration curves across primary restorative material subgroups (Zirconia vs. PFM). **(E)** Empirical sensitivity vs. (1—specificity) trade-off curve reflecting stable multi-class discriminative capacity. **(F)** Comparison of relative feature importance across restoration materials, confirming that Margin Fit and Screw Access Space remain the top contributors irrespective of prosthesis type.

### 3.7 Implementation workflow and consistency

To facilitate translational integration, a clinical implementation pipeline was structured (Figure 7). The risk probability heatmap illustrates the compounding effect between vertical marginal gaps and restricted screw access space (Figure 7A). Subgroup forest plots confirmed stable discriminative performance across anatomical positions and restorative materials, with AUCs consistently maintaining between 0.78 and 0.85 (Figure 7B). The standardized workflow guides practitioners through prosthodontic data input, automated ML inference, and tiered decision support (Figure 7C). Agreement analysis between the AI model and senior clinical specialists showed an acceptable correlation of *r* = 0.76 (Figure 7D, Table 5). Based on the calculated prosthetic hazard tiers, personalized maintenance recall schedules were formulated to monitor high-risk sites proactively (Figure 7E), with visual scoring points directly convertible for chairside use (Figure 7F).

**Figure 7:**
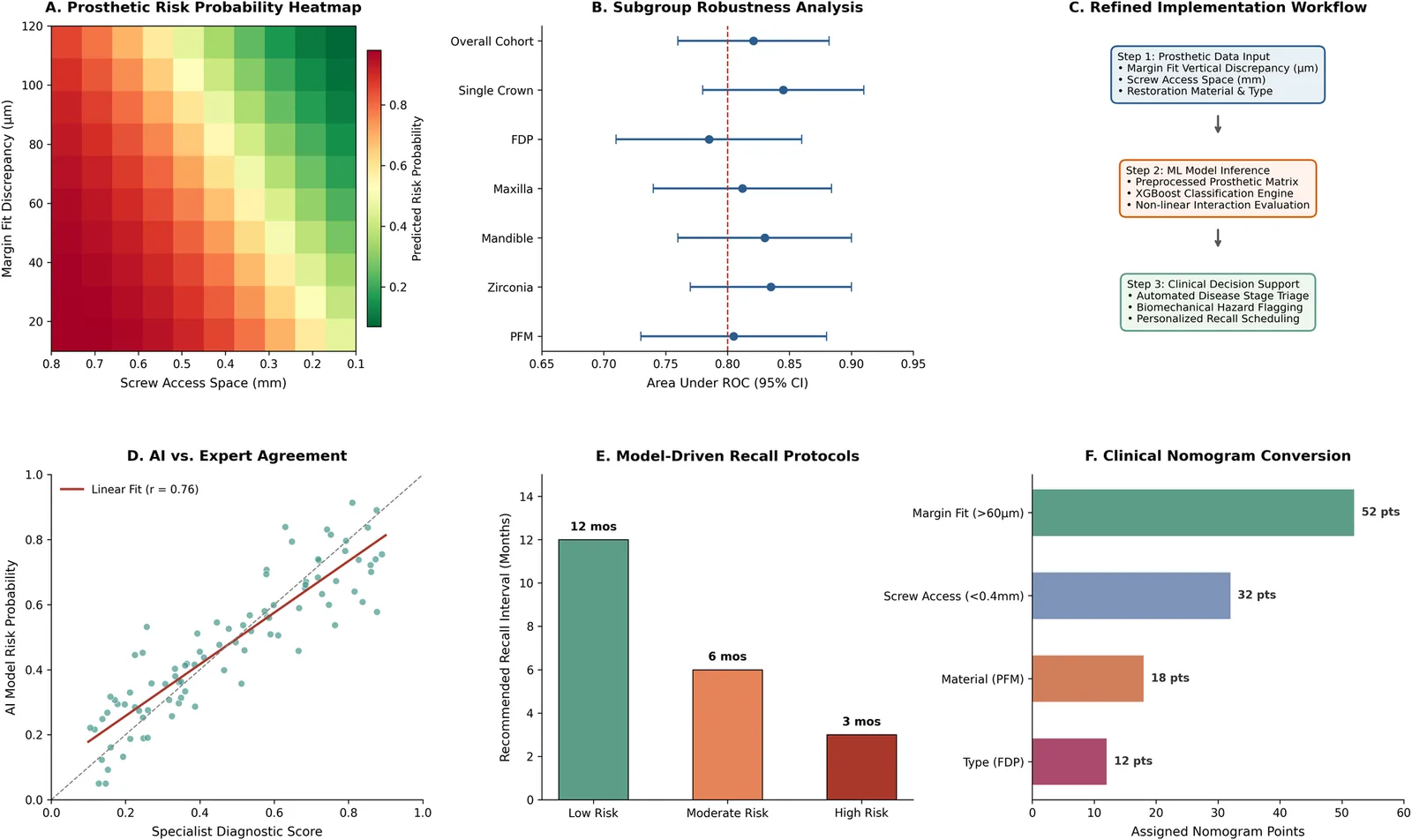
Clinical implementation framework, sub-cohort stability, and translational decision logic. **(A)** Risk probability heatmap mapping combinations of Margin Fit discrepancies and Screw Access Space constraints to predicted disease classification probabilities. **(B)** Forest plot illustrating subgroup classification stability across clinical covariates (implant location, prosthesis design, and material). **(C)** Refined three-stage implementation workflow: Step 1 (raw prosthodontic data acquisition), Step 2 (ML model inference via the XGBoost engine), and Step 3 (personalized clinical decision support). **(D)** Agreement between AI-predicted disease probabilities and senior specialist clinical assessments (*r* = 0.76). **(E)** Tailored recall schedules stratified by prosthetic hazard tiers (Low risk: 12 months, Moderate risk: 6 months, High risk: 3 months). **(F)** Translation into nomogram point weighting based on multivariable ordinal logistic regression.

**Table 5:** Inter-rater and diagnostic consistency between the prosthodontic AI model and clinical diagnosis on the validation cohort (*n* = 90).

Comparison pair	Cohen’s quadratic weighted *κ*	Overall diagnostic agreement (%)	Kendall’s tau-b (τb)
AI model vs. senior specialist 1	0.762	78.9%	0.725
AI model vs. senior specialist 2	0.748	77.8%	0.710
Specialist 1 vs. specialist 2	0.841	85.6%	0.804

### 3.8 Comprehensive impact and diagnostic gain

Advanced metrics and human-machine comparisons were used to assess practical value (Figure 8). PR analysis demonstrated robust average precision for peri-implantitis identification (Figure 8A). Learning curves confirmed that the model reached a performance plateau around 200 samples, indicating adequate training sample size (Figure 8B). In comparative benchmarking, AI-assisted evaluation significantly improved diagnostic staging consistency compared to clinicians alone (*p* < 0.001, Figure 8C). Finally, the model-driven intervention was projected to optimize clinical management in 90% of all cases, facilitating earlier diagnosis and optimized treatment planning (Figure 8D).

**Figure 8:**
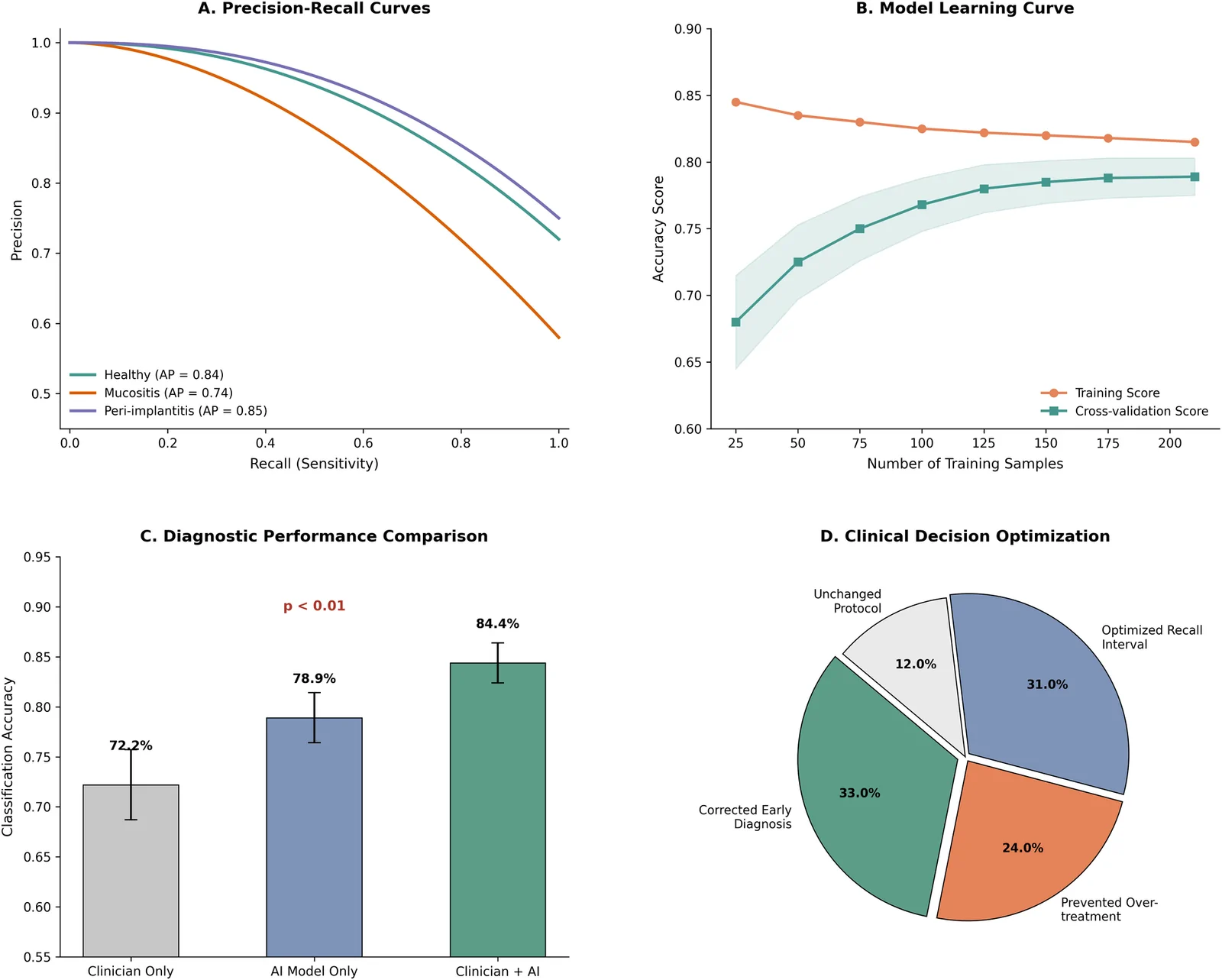
Longitudinal impact and human-machine diagnostic comparison. **(A)** Multi-class Precision-Recall curves on the internal validation cohort (Average Precision: 0.84 for Healthy, 0.74 for Mucositis, and 0.85 for Peri-implantitis). **(B)** Model learning curves demonstrating cross-validation convergence as training sample size expands to *n* = 210, confirming adequate sample size without overt overfitting. **(C)** Head-to-head classification accuracy comparison between clinicians alone (72.2%), AI model standalone (78.9%), and hybrid human-in-the-loop triage (Clinician + AI: 84.4%, *p* < 0.01). **(D)** Pie chart illustrating the projected distribution of model-assisted clinical decision refinement, including early detection correction (33.0%) and optimized maintenance scheduling (31.0%).

## 4 Discussion

In this study, we successfully developed and validated a machine learning model incorporating multi-dimensional prosthodontic and clinical features to predict the severity of peri-implant diseases. The XGBoost model demonstrated excellent performance, with high accuracy, AUC, and well-calibrated probabilities, outperforming both other machine learning algorithms and a traditional multivariate logistic regression model. Through SHAP analysis, we enhanced the model's interpretability, and the development of a nomogram and DCA confirmed its potential clinical utility. Our findings underscore the power of integrating detailed prosthodontic parameters with standard clinical indicators to achieve a more precise and automated assessment of peri-implant health.

While clinical parameters like Probing Depth and Bleeding Index are universally recognized as the cornerstone for establishing a diagnosis (12), their inclusion in predictive algorithms introduces circular reasoning. By deliberately excluding them from the input matrix, our results highlight the significant and independent contribution of purely prosthodontic configurations. The significant association between vertical Margin Fit discrepancies and advancing disease stages, along with its ranking as the top predictor in our non-linear model, provides quantitative evidence supporting the clinical observation that restoration microgaps represent critical mechanical correlates of peri-implant pathology. This aligns with the findings of Jafer et al. and others who have emphasized the biological impact of microgaps and cement excess (13–15). Similarly, Screw Access Space emerged as a novel but relevant predictor, likely reflecting the ease of cleaning and the potential for microbial colonization in the prosthetic components (16–18). By moving beyond purely clinical signs, our model captures the iatrogenic and biomechanical aspects of implant therapy, leading to a more holistic risk assessment. Importantly, given the retrospective, cross-sectional design of this study, the observed relationships between prosthodontic features (such as Margin Fit discrepancies and constricted Screw Access Space) and disease classifications must be interpreted strictly as statistical associations rather than proven causal mechanisms. Although microgaps are known to harbor bacterial plaque, longitudinal prospective studies monitoring pristine restorations from baseline are essential to definitively establish temporal causation.

The superior performance of the XGBoost model over traditional logistic regression is noteworthy (19). The univariate and multivariate analyses confirmed the linear relationships of the main risk factors, which were well-represented in the nomogram (20). However, XGBoost's higher accuracy suggests its ability to capture complex, non-linear interactions among features that are not accounted for in standard regression models. For instance, the SHAP interaction plot revealed that the adverse effect of Margin Fit discrepancies was markedly amplified in the presence of a constricted Screw Access Space (<0.4 mm), a nuanced non-linear biomechanical interaction that standard additive linear models fail to capture (21–23). This capability to learn from data without pre-specified interaction terms is a key advantage of machine learning in complex clinical scenarios.

A critical aspect of any predictive model is its interpretability and trustworthiness, which are essential for clinical adoption (24, 25). Our use of SHAP values addressed this problem. The SHAP summary plot provided an intuitive, global view of the model's logic, reinforcing the predominant biomechanical impact of Margin Fit and Screw Access Space while quantifying the relative contributions of restorative materials (26, 27). The local force plot offered a transparent explanation for an individual prediction, which could be a powerful tool for patient-clinician communication. This level of transparency is a significant step forward from opaque models and is increasingly demanded by both clinicians and regulatory bodies (28–30).

To bridge the gap between the complex algorithm and point-of-care use, we developed a simple nomogram. While its performance was slightly lower than the full XGBoost model, its ease of use makes it a valuable tool for initial screening, especially in settings without advanced software. Furthermore, the Decision Curve Analysis provided compelling evidence for the model's clinical net benefit, showing that it could guide decision-making more effectively than standard “treat-all” or “treat-none” approaches across a wide range of clinical thresholds.

This study has several important methodological limitations. First, we explicitly emphasize that the evaluation conducted herein represents an internal hold-out validation rather than a true external validation, even though strict patient-level separation was maintained to avoid intra-subject bias. Because data were collected from a single medical center, subtle institutional biases in implant placement and prosthetic workflows cannot be excluded, and multi-center prospective studies using external validation cohorts are required before claiming broad generalizability. Second, regarding sample size and dimensionality, while the revised feature set (5 predictors) yielded an improved EPV of approximately 14.0 for the peri-implantitis category and cross-validation learning curves showed minimal divergence, mild risk of model overfitting cannot be entirely ruled out in tree-based architectures. Third, while we included detailed prosthodontic features, several critical patient-level systemic modifiers (e.g., precise plaque index, smoking habits, and glycemic control) were unavailable in retrospective charts. Finally, 2D periapical radiographs were used to assess Margin Fit and Screw Access Space, which may provide less comprehensive spatial resolution than 3D cone-beam computed tomography (CBCT).

Future research should focus on external validation of this model in multi-center studies to test its transportability and robustness. Prospective trials are needed to assess its real-world impact on clinical outcomes, such as early detection leading to improved treatment success rates. Incorporating additional patient-level risk factors and leveraging more advanced imaging data could further enhance the model's predictive power. Ultimately, the integration of such a model into clinical decision-support software could revolutionize the management of peri-implant health, enabling a shift from a reactive to a proactive, personalized, and precision-based approach to care.

## 5 Conclusion

In conclusion, this study demonstrates that a machine learning model, particularly XGBoost, can accurately predict the severity of peri-implant diseases by integrating both standard clinical measurements and detailed prosthodontic features. Key contributing features include Margin Fit vertical discrepancy, Screw Access Space, and restorative material. The model's high performance, combined with its interpretability via SHAP and its demonstrated clinical utility through DCA and a user-friendly nomogram, positions it as a promising tool for enhancing the precision of peri-implant disease diagnosis and management.

## Data Availability

The datasets presented in this article are not readily available because the dataset contains sensitive clinical records and multi-dimensional longitudinal parameters of individual patients, including dental implant characteristics, exact clinical probing measurements, and radiographic evaluations. According to the strict data protection policies of the Institutional Review Board of the Second Hospital of Hebei Medical University and national medical privacy regulations, public dissemination of this raw repository is prohibited to prevent any potential re-identification or breach of patient confidentiality. Consequently, data sharing is restricted to non-commercial, academic verification purposes only, and will be made available upon reasonable request following secondary ethical vetting. Requests to access the datasets should be directed to Name: Guanjun Zhou 28111741@hebmu.edu.cn.
